# Confidence and motivation to help those with a mental health problem: experiences from a study of nursing students completing mental health first aid (MHFA) training

**DOI:** 10.1186/s12909-020-1983-2

**Published:** 2020-03-06

**Authors:** Gemma Crawford, Sharyn Burns

**Affiliations:** Collaboration for Evidence, Research and Impact in Public Health, School of Public Health, Faculty of Health Sciences, Curtin University, GPO Box U1987, Perth, 6845 Western Australia

**Keywords:** Mental health first aid, Mental health, Universities, Nursing students, Prevention, Early intervention, Motivators, Training, Education, Mental health literacy

## Abstract

**Background:**

Those studying nursing are at greater risk for developing mental health problems than other tertiary students. Mental Health First Aid (MHFA) training may assist students to support peers and build mental health literacy. Understanding motivation to participate in training can identify factors influencing uptake and completion. This paper explores motivators for university nursing students to participate in MHFA training and uses previous experience and confidence in assisting someone with a mental health problem to triangulate data.

**Method:**

A randomised controlled trial was employed to measure the impact of the course for nursing students at a large Western Australian university. An online survey was administered prior to MHFA training with undergraduate nursing students (*n* = 140). Thematic analysis of open-ended questions explores motivators to participate and help provided to an individual. Baseline frequencies describe demographics, confidence in helping and exposure to someone with a mental health problem. A Chi Square test compared confidence in helping and exposure to someone with a mental health problem.

**Results:**

More than half of participants reported contact with individuals experiencing mental health problems (55%; *n* = 77); approximately a third (35.8%) reported limited confidence to assist. Those in previous contact with someone with a mental health problem (71.5%; *n* = 55) were significantly more likely to feel confident in helping (*p* = 0.044). Mental health literacy, helping others, career and experiences were described as training motivators.

**Conclusion:**

Exploiting motivators, both intrinsic and extrinsic may increase MHFA training uptake and completion. Tertiary institutions would benefit from policy to embed MHFA training into nursing degrees. The training may have utility for university degrees more broadly.

**Trial registration:**

Australian New Zealand Clinical Trials Registry; ACTRN12614000861651. Registered 11 August 2014 (retrospectively registered).

## Background

Each year, around 20% of Australians aged 16 to 85 will experience a mental health problem [1]. Of those affected, 75% will experience their first episode by age 25 [1]. Despite high prevalence and early onset, help seeking remains low [2–4] and mental health literacy remains inadequate [5, 6]. Improving community mental health literacy may encourage professional help seeking, reduce stigma, and increase support networks [7–11].

Internationally, poor mental health and subsequent impacts is recognised as a concern for tertiary institutions [12–15]. Studies suggest tertiary students have an increased likelihood of experiencing psychological distress compared to non-students their age [16–19]. Further, research has shown young people do not seek professional help for their mental health problems [14, 20], with students more likely to confide in close friends or family or choose self-help strategies instead of counselling [20, 21]. Mental health problems among university students can lead to negative impacts on academic success including cessation of studies, high levels of absenteeism and failure, and reduced productivity [12, 22–25]. Attending university may increase levels of distress for many young people, consequently appropriate support is required to throughout studies and in the transition to the workforce [26–28].

The demands of studying specific courses including nursing have been associated with stressors that can result in additional distress as students advance through the course and increase risks for developing mental health problems [27–31]. An Australian study of undergraduate nursing students (*n* = 431) found exhaustion and stress levels increased each year [32]. Research by Evans and Kelly support these findings, suggesting additional challenges for nursing students including academic workload, theory–practice gap, difficulties and realities of clinical placement, leading to feeling overwhelmed and a range of short term coping mechanisms to deal with exhaustion and stress [33].

In addition to personal mental health risks, poor levels of mental health literacy are found amongst nursing students in the formative component of their degrees [34]. Ongoing educational training has been associated with enhanced positive attitudes among nursing students towards the mental health sector [35]. This is critical as in Australia, nurses represent around half the mental health workforce [36–38].

Mental Health First Aid (MHFA) is a training course, facilitating skills to assist those experiencing a mental health problem or mental health crisis [10, 39]. The course is designed to build mental health literacy, reducing stigma and discrimination towards mental health and providing direction for supportive actions [4, 40, 41]. Since the program’s inception, extensive evaluation has consistently suggested a range of participation benefits. A systematic review of research related to MHFA training concluded that in all studies, MHFA training demonstrated increased mental health awareness and knowledge amongst participants [42]. Other positive outcomes included: improved recognition of mental illnesses [27, 42–47];, reduced stigmatising attitudes [9, 27, 39, 42–44, 47–49] and decreased social distance [27, 46, 47, 49, 50]. Additionally, probability of recommending professional help increased [39, 42, 46, 49, 50] and participants developed greater confidence to assist individuals experiencing a mental health problem [9, 27, 39, 43, 44, 46, 48–50]. Importantly, these outcomes have been maintained after course completion [39, 51].

In 2012, MHFA Australia commenced offering tailored courses to nursing students as part of an initiative to train ‘frontline workers’ [52]. The training aimed to benefit personal mental health and provide knowledge and skills to be used with peers and other adults**.** While the stated aim of the training was not mental health training [29], it may be that professional development is a motivation for many nursing students to participate.

Understanding motivation is important to identify factors that may influence why individuals choose to complete the course. Motivation can be described as either intrinsic (e.g. perceived internal and personal rewards for completing a task or action, such as increased knowledge or sense of accomplishment or self-worth) or extrinsic (e.g. external rewards, such as receiving recognition or good marks or avoiding punishment) [53, 54].

Studies suggest that despite the emphasis on external recognition in many institutions, intrinsic factors are often a key motivator for students studying at university [53, 55]. Intrinsic motivation is seen as having meaningful and positive cognitive engagement with an individual’s learning [53]. The ability to link current course work to future benefits and long term goals has been found a highly motivating, intrinsic factor [55] for high school or university students [56, 57] and may influence why individuals complete extracurricular courses.

Accordingly, MHFA training may provide a useful addition to curricula [45, 58, 59] to assist undergraduate nursing students to meet the stated aim of the course to increase knowledge and skills relating to mental health problems, reduce stigma towards individuals or peers with mental health problems [28, 29] and support peers. However, students may also be motivated to take part in MHFA training due to additional benefits consistent with those described above relating to community contribution and career advancement.

This study was part of a broader research project to determine the impact of Mental Health First Aid training on pre-service university nursing students and the factors influencing participation [28]. The broader study measured outcomes including: knowledge, recognition of depression, first aid intentions and stigmatising attitudes (published elsewhere) [60]. The aim of the research presented in this paper was to describe the participant profile and explore personal motivation of first year nursing students to participate in MHFA training. Open-ended questions explore motivators for participation and data are triangulated with demographics, previous experience and confidence in assisting someone with a mental health problem. Findings will inform Mental Health First Aid and other training interventions for nursing students in a university setting.

## Methods

The broader study employed a pragmatic, waitlisted, randomised controlled trial (methods described briefly below and in detail elsewhere [28, 60]). For the present study, survey data collected at baseline (quantitative and qualitative) was used to explore pre-service nursing student motivators for completing MHFA training and to investigate key demographics, previous mental health training, experience in helping someone with a mental health problem and perceived confidence to help prior to completing the training.

### Procedure

Participants were eligible to participate if they were undergraduate, first-year nursing students aged 18 years and over enrolled via internal teaching mode at a large Australian university. A total of 200 nursing students registered for the intervention. After initial drop out and removal of ineligible students (*n* = 19) (those who did not meet the eligibility described above), 181 students were randomised into either the intervention (*n* = 92) or control (*n* = 89) group. The intervention group participated in tailored MHFA training [28]. The control group received no intervention during the data collection period, but was waitlisted to be offered online MHFA training on intervention conclusion. Baseline quantitative and qualitative data were collected from intervention and control group participants via a self-complete online survey administered via student email 1 week prior to the commencement of the training course. The sample and procedure as it relates to the CONSORT guidelines is described in detail elsewhere [28, 60]. Ethical approval was granted by the Curtin University Human Research Ethics Committee (SPH-74-2013). This trial was registered with the Australian New Zealand Clinical Trials Registry: ACTRN12614000861651.

### Survey

The survey was based on previous questionnaires developed to measure mental health literacy, confidence, MHFA intentions and stigmatising attitudes [27, 43, 44, 46, 47, 50, 61, 62]. The following items were collected at baseline and are described in this paper: previous completion of MHFA or other training, units or placements; motivation for undertaking MHFA training; confidence to assist someone with a mental health problem; contact with someone with a mental health problem; and previous experience in providing help to someone with a mental health problem. Table 1 describes the items included in the survey and where they have been reported. Instrument development is described in detail elsewhere [28, 60].

**Table 1:** Survey Items

Survey Domains	Items
**Demographics** (collected at baseline)	Gender, age, student status (domestic/international), enrolment status (full time/part time)
**Previous experience with mental health** (collected at baseline and reported here)	To determine familiarity with course content and previous mental health skills and knowledge, participants were asked if they had previously completed MHFA training, other education or training in mental health, mental health units in their current course of study or a mental health related clinical placement during current course of study. Responses were dichotomised to ‘yes’ or ‘no’. Participants who had completed other training, units or relevant placements, were asked to provide details including the provider and the year completed.
**Motivation for undertaking MHFA training** (collected at baseline and reported here)	Assessed using an open ended question was used to explore motivation for completing the MHFA course [46, 50, 63, 64].
**Existing confidence to assist someone with a mental health problem** (collected at baseline and reported here)	Assessed by asking participants if they felt confident in helping someone with a mental health problem. A five point Likert scale was provided with responses ranging from ‘not at all’ to ‘extremely confident’ [46, 50].
**Previous contact with someone with a mental health problem** (collected at baseline and reported here)	To determine exposure to individuals with mental health problems participants were asked if they had had contact with someone with a mental health problem during the last six months. Responses included *‘yes’*, *‘no’* and *‘don’t know’*.
**Previous experience in providing help to someone with a mental health problem** (collected at baseline and reported here)	Two questions asked of those who answered yes to previous contact. 1. Open ended question which asked how many people with a mental health problem they had contact with during this period. Coded to a few (1–3), some (4–9) and many (10 or more). 2. The second question asked if they offered any help. Responses included *‘not at all’*, *‘a little’*, *‘some’* and *‘a lot’*. These questions were followed with a qualitative question regarding what type of help was provided. These questions have been used in previous evaluations of MHFA training [46, 50]. Responses were coded against 10 broad categories with one point given for each response provided using the scoring described by Kelly and colleagues [9].
**Mental health knowledge** (collected at all timepoints and reported previously)	Knowledge was assessed using 20 true or false statements adapted from previously validated MHFA research. Recognition of depression was assessed using a specific vignette about “John” and an open ended question “what, if anything is wrong with John?” which has been used in previous research.
**Confidence and Intentions** (collected at all timepoints and reported previously)	Confidence was assessed by asking participants to rate their confidence to help ‘John’. Responses included a five point Likert scale ranging from: don’t know to very confident. This measure has been used in previous MHFA research. Mental health first aid intentions were assessed using an open-ended question “Imagine John is someone you have known for a long time and care about. You want to help him. What would you do? Responses were scored using a previously used system in other MHFA studies and based on the ALGEE action plan which is a key focus of MHFA.
**Stigma** (collected at all timepoints and reported previously)	Stigmatising attitudes including social distance were measured. Stigmatising attitudes were assessed using a seven item scale adapted from the validated Depression Stigma Scale to measure personal and perceived stigma. Social distance was measured by a scale adapted from the validated Social Distance Scale. Both have been used in other MHFA studies.

### Data analysis

Frequencies were computed to describe demographics, confidence in helping and exposure to someone with a mental health problem. Proportions were compared for confidence in helping and exposure to someone with a mental health problem using a Chi Square test. Statistical significance was determined at *p* < 0.05. Quantitative data were analysed using SPSS version 22 [65]. Qualitative data were collected via open-ended responses to questions in the online survey. Motivators for participation and help provided to an individual with a mental health problem were explored via Braun and Clarke’s broad approach to thematic analysis [66] and consistent with thematic analysis in other qualitative studies by the research team [67–70]. Quantitative data were triangulated with qualitative data to enhance credibility. Thematic analysis incorporated the following steps: 1) transcribing and becoming familiar with the data which involved reading and re-reading transcripts; 2) generating a broad list of initial codes by examining words and phrases of each response; 3) collating codes into potential themes; 4) reviewing themes and creating a thematic map; 5) defining and refining core themes and sub-themes; here mental health literacy was positioned as the central, underpinning theme 6) selecting exemplar quotations for inclusion in analysis and relating analysis to research aims. Both members of the research team independently coded data. Examination of data by multiple researchers reduced bias and improved confirmability of the study [71]. Qualitative data was managed using QSR International’s NVivo 11 Software [72].

## Results

### Group characteristics

Of the 140 participants, 83.6% (*n* = 117) were female. Approximately three quarters (75.7%, *n* = 106) of the sample were aged 18 to 24 years. The majority (97.1%, *n* = 136) had no previous mental health training prior to the course. Demographics are summarised in Table 2.

**Table 2:** Participant characteristics and previous mental health experience

Demographic	Total (%)
**Gender**	
Male	23 (16.4)
Female	117 (83.6)
**Age**	
18–24	106 (75.7)
25–30	16 (11.4)
31–35	7 (5)
36–40	3 (2.1)
41+	8 (5.7)
**Mental health units completed during degree**	
Yes	6 (4.3)
No	134 (95.7)
**Practical mental health facility placement during degree**	
Yes	2 (1.4)
No	138 (98.6)
**Confidence in helping someone with a mental health problem**	
Not at all	11 (7.9)
A little bit	39 (27.9)
Moderately	50 (35.7)
Quite a bit	33 (23.6)
Extremely	7 (5)
**Contact with someone with a mental health problem (past 6 months)**	
Yes	77 (55)
No	45 (32.1)
Don’t know	18 (12.9)
**Number of people in contact with a mental health problem**	
A few (1–3)	48 (62.3)
Some (4–9)	18 (23.4)
Many (10+)	11 (14.3)
**Support for someone with a mental health problem (if contact) (*n*** **= 77)**	
Not at all	3 (3.9)
A little	26 (33.8)
Some	26 (33.8)
A lot	22 (26.8)

### Previous mental health training

Eleven participants had undertaken some form of prior training in mental health. Three participants had previously undertaken mental health training/education in a workplace environment. Six participants had undertaken previous mental health units in their nursing degree; three were specific mental health units, while the others included mental health as a component. Two participants had undertaken practical placements in a mental health facility. These placements were in nursing homes and ranged from 6 days to 3 weeks.

### Confidence

Prior to course participation, confidence in helping someone with a mental health problem varied. Around a third of participants reported being ‘quite’ or ‘extremely’ (30.6%; *n* = 40); confident while a further third reported having ‘little’ or ‘no’ confidence (35.8%; *n* = 50) in helping someone with a mental health problem (see Table 1).

### Previous experiences with mental health

Over half (55%; *n* = 77) of participants reported direct contact with an individual experiencing a mental health issue within the preceding 6 months while 32.1% reported no contact and around 13% were unsure if they had come into contact with someone in the preceding 6 months. Two-thirds of these participants indicated they had had contact with a few (62.3%; *n* = 48) people with a mental health problem during this time period. Participants who had contact with someone with a mental health problem were significantly more likely to feel confident in helping compared to those who reported no contact (χ 2 = 12.952; df = 6; *p* = 0.044). Of those who had had contact the majority (71.5%; *n* = 55) felt moderately to extremely confident in helping compared to 57.8% (*n* = 26) of those who had not come into contact with someone with a mental health problem.

### Previous type of help offered

Of the 77 participants who reported direct contact with an individual experiencing a mental health problem, almost all had attempted to offer some help. The majority (67.6%; *n* = 52) had offered ‘a little’ or ‘some’ help.

Of those reporting help across ten broad categories (see Table 3), just over three quarters (*n* = 58) provided a response in the *“Other”* category such as: *“Assisting them on public transport”* and *“Offering friendship and help with English.”* Helping consistent with the nine categories (below) were broadly: *“spent time listening to their problems”* (50.7%; *n* = 39); *“recommended they seek professional help”* (14%; *n* = 11) or *“gave them information about the problem”* (11.7%; *n* = 9). The following quote highlights these types of help:

**Table 3:** Ten categories of help offered

Category of help offered	Total (%)
1. Spent time listening to their problem	39 (50.64)
2. Helped to calm them down	4 (5.19)
3. Talked to them about suicidal thoughts	1 (1.29)
4. Recommended they seek professional help	11 (14.28)
5. Recommended self-help strategies	5 (6.49)
6. Gave them information about their problem	9 (11.68)
7. Gave them information about local services	3 (3.89)
8. Made an appointment for them with services	4 (5.19)
9. Referred them to books or websites about their problem	0 (0)
10. Other	58 (75.32)

“I’ve said they can talk to me, referred them to counselling (headspace, kids’ helpline, lifeline etc), offered them assistance when they need help, asked to take them out to get their mind off things … ”

Around two in five participants (38.9%; *n* = 30) had provided help across two categories. Just under half (44.1%; *n* = 34) had provided help in only one category while just over 10% had provided assistance across three categories (13%; *n* = 10). Few participants had offered help across four (2.6%; *n* = 2) or five (1.3%; *n* = 1) categories.

### Motivators to complete MHFA training

**Fig. 1:**
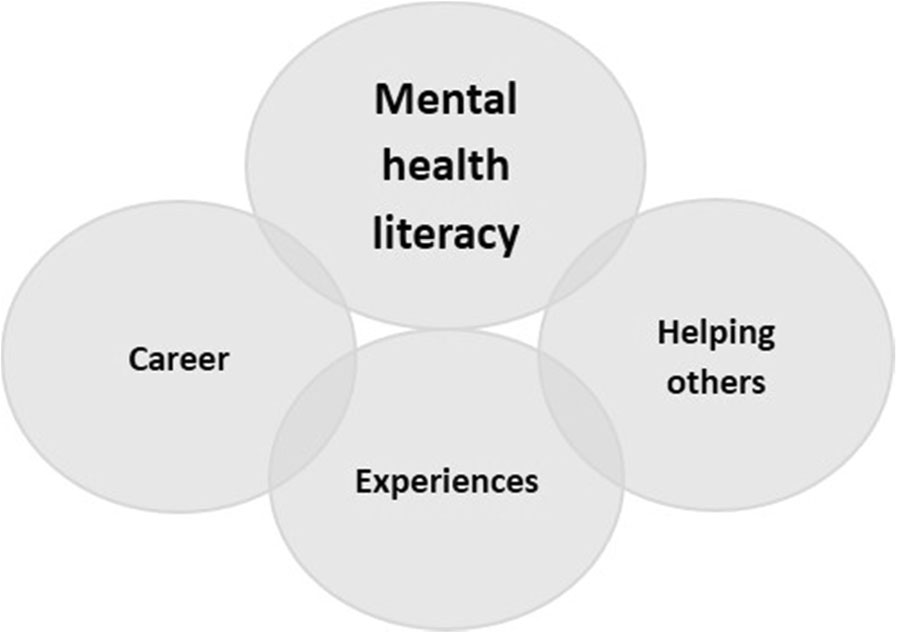
Motivation to participate in Mental Health First Aid

Motivators for completing MHFA training were described through the four interrelated key themes of mental health literacy (knowledge, confidence and skills), helping others (described in relation to those who may be experiencing a mental health problem or crisis), career (improved skills or advancement) and experiences (described in relation to personal experiences of family, friends, peers and own mental health) (see Fig. 1 below).

Improving mental health literacy was an underlying motivator that was strongly linked to all themes. Helping others was linked to career while experiences influenced the desire to help others, and for some, motivated career choices.

The majority of participants described wanting to improve their mental health knowledge, skills and confidence (mental health literacy). Many of these comments reflected a desire to improve aspects of mental health literacy as they felt this would enhance their career as a nurse (career) but would also provide useful skills for everyday life (helping others). The importance of the need to enhance skills for their career and to help others generally is highlighted by the following:

“I believe dealing with mental illnesses will be a major part of my career as a nurse. Not only with my patients but also with the people around me in life and even within my family and close relationships. This course will help me to understand metal health better and teach me how to deal with certain situations and circumstances and how I can best offer myself to help those people.”

While many of the comments reflected an initial intrinsic desire to enhance skills so they were more competitive in the workforce, or to complete voluntary hours as part of their degree, participants also reflected on the importance of the course in enhancing skills with a more extrinsic motivation. Some participants identified a lack of confidence in working with and being around people with mental health issues. Others identified the importance of the course in improving their skills in working with people with mental health problems, both in a professional situation and in everyday life. The participant statement: “*To improve myself as a person and be a better health professional”* provides a succinct representation of much of the discussion. The relationship between the career, mental health literacy and helping others themes is highlighted by the following:

“It's a great opportunity for further education. I think that having a recognised certificate will help set myself apart from others when applying for graduate positions. I also lack confidence when dealing with mental health so an early insight will be helpful.”

The initial motivation of completing voluntary hours as part of their degree was expressed by some participants, however all participants who discussed the benefit of completing the course for volunteer hours also recognised the benefits of the course in terms of enhancing mental health literacy. Few participants identified that the course was free and that this was a motivator for participation:

“My motivation originally was to gain a few hours for my clinical log for [my unit] but since I have read more about this course and I feel it will benefit my skills when I become a nurse.”

Helping others was a strong motivator for participants to complete this course. While some participants discussed the need to enhance mental health literacy to enhance their career, others expressed a desire to enhance skills to help others who may be suffering a mental health problem or crisis, for example:

“The idea that I will be in a position to help someone in a mental health crisis and be able to improve my skills.”

While some respondents who discussed helping others referred to helping people generally, the theme of experiences linked closely to the need to help. Participants reflected on their own personal experiences of family, friends, peers and their own mental health problems and the desire to learn more about mental health problems and to enhance skills in dealing with issues. A few participants, typified by the following quote, discussed their passion for mental health which had developed as a result of personal experiences:

“I have an extreme passion and interest in mental health, and feel this is from growing up with a family member who has lived a life with mental health issues. I want to learn more about how to help them.”

Others expressed a desire to better understand mental health and to help friends and peers who were experiencing mental health problems:

“To be able to help someone with a mental illness, to better understand them as I have had a few friends suffer severe depression and felt there wasn’t anything I could do to help.”

A number of participants reflected on their own experiences of poor mental health which generated a desire to help others, for example, “*I have my own mental health issues and would like to help others”*. Some reflected that even though they had personally experienced mental health problems they lacked knowledge:

“In the past I have been through some mental illnesses and so my main motivation for completing the Mental Health First Aid course is so I can gain more knowledge about signs and symptoms of most mental illnesses, so I can help them out as soon as I can before professional treatment is received or the crisis resolves.”

## Discussion

This paper explored motivation for nursing students to participate in tailored MHFA training. To better understand participant characteristics, the research measured levels of pre-training past experience and confidence relating to assisting someone with a mental health problem. Four interrelated themes including *mental health literacy*; *helping others*; *career*; and *experiences* emerged to understand motivators to participation in the training.

The majority of participants had not previously participated in any mental health training, education or placements and articulated a desire to enhance their *mental health literacy*., Consistent with the themes of *helping others* and *experiences* more than half reported direct contact with an individual experiencing a mental health issue within the previous 6 months. Those in previous contact with an individual with a mental health problem were significantly more likely to feel confident in helping compared to those who reported no contact. This higher level of confidence may be due to the nursing specific sample who have had some opportunity to put mental health knowledge in to practice [58], however is also consistent with findings in other MHFA studies relating to nursing students [27, 45, 58], other health disciplines [47, 73] and broader university studies [74].

Of those who reported contact with an individual experiencing a mental health problem almost all had attempted to offer some form of help. Around half of participants specifically indicated that they had *“spent time listening to their problems”*. This finding is consistent with previous MHFA research, specifically a randomised controlled trial which found at 2 year follow up the most commonly reported type of help offered was “listening non-judgementally” [75]. Participants discussed their desire for enhanced *mental health literacy* to enable them to better *help others*. Findings suggest those with a little previous experience may be motivated to attend training in recognition of the need to enhance their mental health first aid skills.

Themes presented in this study are consistent with those in other studies that describe participant motivation to take part in MHFA training. The literature suggests that motivation to participation may be due to work related reasons [46, 50, 63, 64], knowing someone close who had or was experiencing a mental health problem [46, 50], personal mental health [46, 50], because they believed it their duty to learn the appropriate skills [46, 50, 63] or for general knowledge [46, 50, 63, 64]. Additionally, a study conducted among MHFA instructors suggested that personal experiences, working in mental health and volunteering were motivators for completing training [76]. It has been posited that recognition of the need to increase confidence may be another motivator for completing the course [30].

Themes are also consistent with the broader historical literature, such as work by Murphy and colleagues [77] citing a range of studies [78–81] highlighting influential factors in motivating participation in continuing professional development including: personal motivational orientation, improvement of professional knowledge and skills or aspirations for increased professional competence or promotion, and personal growth.

Our research suggests both intrinsic and extrinsic reasons for participation, with interrelated themes highlighting the synergies between both. Mathieu and Martineau [82] suggest several types of training participation motivation. This includes the intrinsic like-‘motivation to learn’ (i.e aspiration to learn content impacts on how much an individual learns during training) and extrinsic like-‘motivation through expectation’ (i.e. expectancy that putting in effort during training will result in skills and knowledge leading to valuable outcomes).

### Implications

We suggest several implications for policy and practice. Happell and colleagues [58] suggest that availability of MHFA training should not negate the need for quality, mental health content in nursing university degrees. However, given the importance of ongoing professional development and reported negative attitudes towards mental health nursing as a career choice [35], implementation of MHFA training early in nursing degrees may contribute to improving desirability of mental health nursing as a future career [59]. This is an additional benefit to ensuring nursing students enhance mental health literacy early in their degree and regardless of the field of nursing that they pursue. Accordingly universities should consider embedding training as a component of courses for all undergraduate nursing students to enhance self and peer support and develop knowledge and confidence for the real world both professionally and personally [45]. Our training indicated the efficacy of delivery in a large, first-year unit with relatively limited resourcing, suggesting it may be a model that has utility for nursing and for health sciences more broadly. Establishing a community of practice of MHFA trained staff and students also has the added benefit of providing peer support to students for present or emerging mental health problems during university study.

The opportunity to better gauge participant motivation and experience prior to training may be useful for academics delivering such training with pre-service nursing students. Individuals motivated to learn are also likely to be motivated to apply acquired skills [82], which is critical if knowledge is to be translated into practice in the workplace and more broadly. Seeking ways to appeal to intrinsic motivation to learn in promotion of training and participation may be valuable by focusing on knowledge and accomplishment [54]. This is an important consideration given findings suggest that intrinsic motivation strongly relates to performance and productivity for adults in the workforce [83]. Linking participation to course learning outcomes and providing credit for participation may be a strategy for consideration.

Consideration of intrinsic motivation can be balanced with appealing to extrinsic motivation. Recognition and reward schemes for MHFA trained staff and students may contribute to motivation to participate and to ensure ongoing currency of knowledge and skills relating to mental health literacy. This may include, developing rewards for course completion (log hours etc) or for performance (such as receiving accreditation that can be added to job readiness skills, making the student more desirable for employment) [84]. For those motivated by the desire to help others, providing opportunities to practice their newly acquired skills by taking on roles as peer champions or mentors within the university environment may be beneficial.

### Limitations

Female participants were overrepresented in this study though this is consistent with the Australian nursing workforce profile [85]. Participants in this study are not representative of the general population. Nursing students likely have better mental health literacy than the general population meaning that results cannot be generalised. However findings may be transferable to other populations of nursing specific or tertiary students more generally. Self-report, survey data was used to capture qualitative responses which limited the ability to seek clarification from participants or undertake member-checking. Interviews may have captured thicker, richer data. Finally, participation and responses may have been influenced by volunteer and social desirability bias which is a limitation of the study.

## Conclusion

This study explored motivators for participation in tailored MHFA training for undergraduate nursing students. The findings highlight the interrelationship between intrinsic and extrinsic motivators with key themes of mental health literacy, career, helping others and personal experience emerging. These findings suggest while intrinsic motivation is important to generate initial interest in completing the MHFA course, extrinsic motivators, especially related to the desire to help others are important in course completion. The prevalence of mental health problems among university students make these motivators very salient. Tertiary institutions would benefit from policy to embed MHFA training into nursing programs to complement existing course material which may also demonstrate utility in other degrees.

## Data Availability

The datasets used and/or analysed during the current study are available from the corresponding author on reasonable request.

## Abbreviation

MHFA: Mental Health First Aid
